# Cuproptosis Signature Would Reveal the Acute‐Remitting Pattern in Patients with Neuromyelitis Optica Spectrum Disorder

**DOI:** 10.1002/advs.202417124

**Published:** 2025-08-14

**Authors:** Peidong Liu, Ruoyu Li, Xinlin Wang, Hui Li, Huiru Hu, Ang Shen, Wenping Zhu, Jiali Pan, Yanmeng Xing, Weiming Xia, Henan Jiao, Di Chen, Lixin Wu, Li Liu, Ju Liu, Bin Yu, Xianzhi Liu, Hongbo Liu

**Affiliations:** ^1^ Department of Neurology the First Affiliated Hospital of Zhengzhou University Zhengzhou University Zhengzhou 450000 China; ^2^ Department of Neurosurgery the First Affiliated Hospital of Zhengzhou University Zhengzhou University Zhengzhou 450000 China; ^3^ Department of Neurology Puyang People's Hospital Puyang 457000 China; ^4^ Department of Neurology Sanbo Brain Hospital Capital Medical University Beijing 100093 China; ^5^ Department of Neurology Zhengzhou Central Hospital Zhengzhou University Zhengzhou 450000 China; ^6^ Department of Neurology the First Affiliated Hospital of Xinxiang Medical University Xinxiang 453100 China

**Keywords:** acute‐remitting course, cuprotosis, NMOSD

## Abstract

The acute‐remitting course (the time interval from remission to the next relapse) is a distinct signature of neuromyelitis optica spectrum disorder (NMOSD), which harms patients and perplexes physicians. However, clinically actionable biomarkers to anticipate NMOSD relapse timing are unavailable. Recently, it is found that Cuproptosis‐related genes would have an impact on the acute‐remitting course in patients with aquaporin‐4 (AQP4)‐immunoglobulin G antibody positive NMOSD. A machine learning‐based algorithm is developed to construct a consensus Cuproptosis‐related prognosis risk (CRPR) model, followed by training a proportional hazards model to divide patients with NMOSD into high/low relapse risk groups. The CRPR is related to the clinical symptoms of patients with NMOSD. Finally, a nomogram is created using CRPR to predict the possibility of relapse in new cases of NMOSD. This prediction model can assist physicians and help develop appropriate interventions to prevent disease relapse.

## Graphical abstract

Neuromyelitis optica spectrum disorder (NMOSD) has distinct acute‐remitting courses but lacks biomarkers for predicting relapse. Recent studies found cuproptosis‐related genes affect its course, and a model built accordingly can predict relapse to aid intervention.

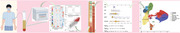

## 1 Introduction

The aquaporin‐4 immunoglobulin G (AQP4‐IgG) antibody positive neuromyelitis optica spectrum disorder (NMOSD) is a non‐fatal but highly disabling disorder, making it harmful for patients. Moreover, attention must be paid to the indeterminate relapse of NMOSD,^[^ ^1^ ^]^ one of its most serious aspects. This affects the patient's physical and psychological health.^[^ ^2^ ^]^ Unlike multiple sclerosis, disability in NMOSD usually occurs secondary to relapses and shows accumulation.^[^ ^3^ ^]^ Based on the 2015 international consensus‐based diagnostic criteria for NMOSD, patients are receiving increasingly precise diagnoses. However, there is no appropriate method to predict the occurrence of a subsequent relapse.

Recently, copper (Cu)‐induced cell death has attracted increasing attention and has emerged as a metabolic regulator of immune tolerance.^[^ ^4^ ^]^ Cu is an essential cofactor in many physiological processes.^[^ ^5^ ^]^ Increased levels of intracellular Cu could also be harmful to the organism, inducing cytotoxicity and even leading to cell death.^[^ ^6^ ^]^ Thus, Cuproptosis, a Cu‐regulated cell death pathway, was discovered.^[^ ^7^ ^]^ Cuproptosis‐programmed cell death is distinct from other processes such as apoptosis, necroptosis, and ferroptosis. Cuproptosis can cause differences in cell death through the tricarboxylic acid (TCA) circle.^[^ ^7^, ^8^ ^]^ Interestingly, the TCA cycle is crucial for the differentiation of naïve T cells,^[^ ^9^ ^]^ including the polarization of regulatory T cells (Tregs)^[^ ^9^ ^]^ and T helper 1 cells (Th1) et al.^[^ ^10^ ^]^ While pathogenic B‐cell‐derived AQP4‐IgG remains the primary effector mechanism in neuromyelitis optica spectrum disorder (NMOSD), emerging evidence indicates that NMOSD pathogenesis involves dysregulated crosstalk between humoral and cellular immunity—not merely humoral dysfunction. For example, recent studies demonstrate that IFN‐γ deficiency initiates NMOSD pathogenesis via an IL‐6/TH17/B‐cell autoreactivity cascade.^[^ ^11^ ^]^ Besides, follicular helper T (TFH) cells drive pathogenic B‐cell activation and antibody production, as demonstrated in our prior work.^[^ ^12^ ^]^ Thus, immunoregulatory networks, particularly T cell‐B cell interactions, would constitute fundamental mechanisms underlying NMOSD pathogenesis. Pathogenic activation of T cells may also explain why there are still cases of relapse after B cell depletion therapy. Cuproptosis could act as a metabolic checkpoint in T‐cell dysregulation, inducing skewed T cell differentiation, particularly proinflammatory subsets, which in turn triggers pathogenic B‐cell activation. Ultimately promotes excessive production of AQP4‐IgG by plasma cells, thereby initiating NMOSD. Therefore, an aberrant T cell immunoregulatory network might be the primary reason for each relapse. Based on those mechanisms, Cuproptosis signatures could quantify the risk of T cell‐mediated relapse. However, can Cuproptosis signatures be used to predict the next relapse of NMOSD in advance? It remains unclear.

This study aims to develop a machine learning based model to predict the relapse possibility of AQP4‐IgG antibody positive NMOSD. We performed a systematic analysis of Cuproptosis based on peripheral blood mononuclear cell (PBMC) mRNA sequencing (RNA‐seq) in patients with NMOSD, followed by the generation of a Cuproptosis‐related prognosis risk (CRPR) model. With this signature, we calculated a risk score for each patient and accessed the period of the acute‐remitting (AR) course related to the risk score. Finally, we developed a nomogram that could help predict NMOSD recurrence. Therefore, in this study, we attempted to develop a Cuproptosis‐based prognostic risk model that could predict the next relapse (the AR course) to inform physicians when to initiate earlier interventions to avoid the next relapse. This procedure could help reduce the accumulating disability and burden in patients with NMOSD.

## 2 Results

### 2.1 Screening of Patients with NMOSD and Collecting PBMC for RNA‐seq

**Figure 1:**
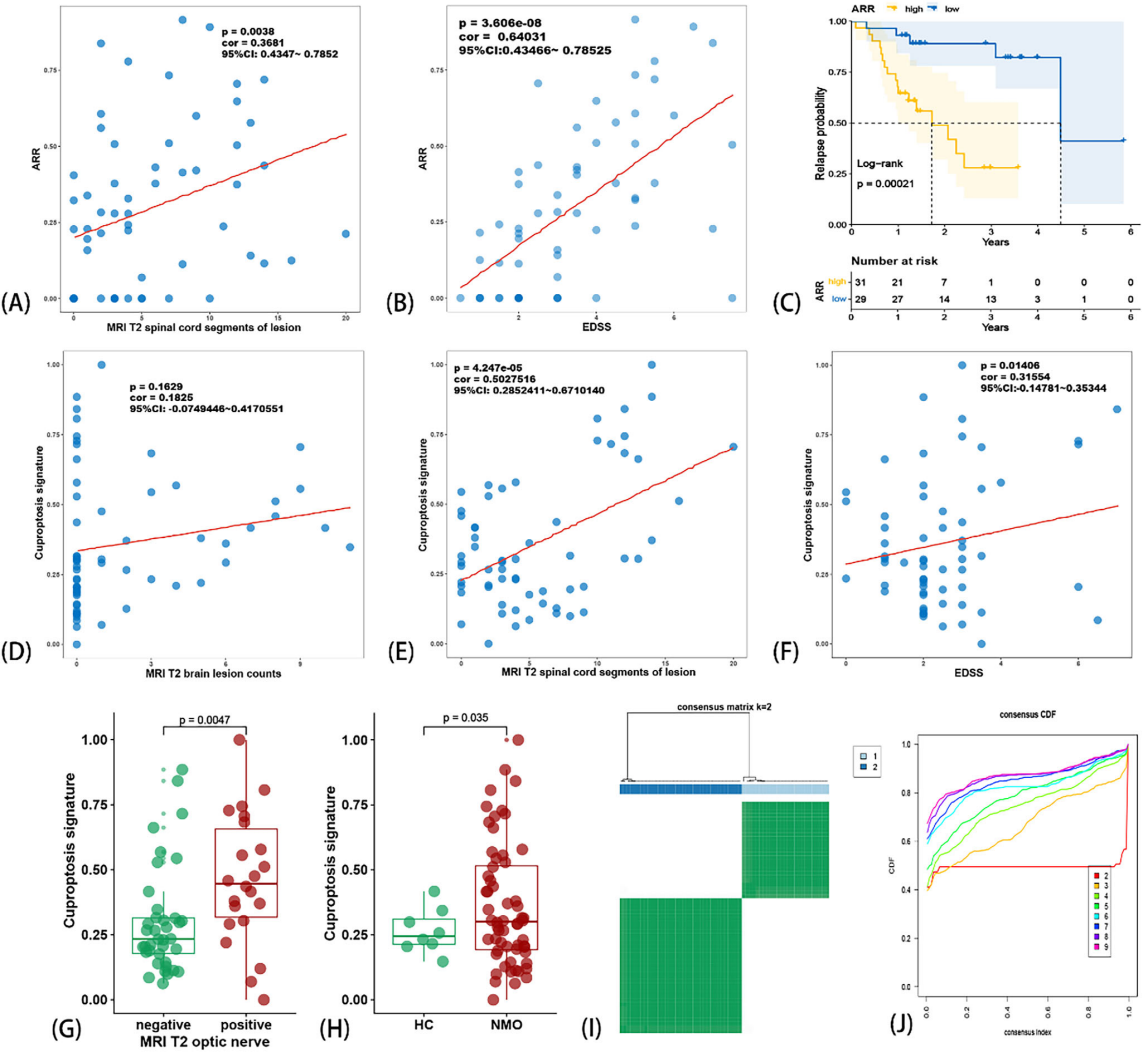
Clinical data display and identification of Cuproptosis signature (CS) with two algorithms. The statistically significant correlation between ARR and counts of spinal cord lesions on MRI T2 weighted images (A) and ARR and EDSS (B). The relapse probability between levels (median) of ARR (C). ssGSEA analysis produced the CS and its correlation between counts of spinal cord/brain lesion on MRI T2 weighted image (D,E). EDSS (F). The CS between positive/negative lesion of optic nerve on MRI T2 weighted image (G) and HC and NMOSD (H). Consensus clustering analysis was used to divide the NMOSD samples into two clusters (I). The Cumulative Distribution Function (CDF) curve of the clusters (J).

All patients diagnosed with NMOSD enrolled in this study were treated at the Department of Neurology of the First Affiliated Hospital of Zhengzhou University (external independent validation cohort was from the First Affiliated Hospital of Xinxiang Medical University, Department of Neurology), and we traced the entire course of each patient with NMOSD up to June 2024. All enrolled samples were positive for AQP4‐IgG antibody and negative for both myelin oligodendrocyte glycoprotein (MOG) ‐IgG and glial fibrillary acidic protein (GFAP) ‐IgG antibody (AQP4‐IgG^+^, MOG‐IgG^−^ and GFAP‐IgG^−^). Besides, the whole samples were collected at the following time points: for patients with NMO in the acute phase, samples were obtained exclusively during the initial 72 h post‐relapse onset and prior to any immunomodulatory therapy (including corticosteroids, intravenous immunoglobulin, or plasma exchange); for patients with NMO in the remission phase (more than 1 month after resolution of acute‐phase symptoms), some patients were prior to admission for biological agent (e.g., monoclonal antibody) therapy, and the others (without biological agent therapy) were sampling when patients returned to the hospital for re‐examination. The general clinical signatures were collected, which included the following: 1) sex; 2) total course of disease; 3) total time of relapse; 4) ARR and clinical signatures based on the time point of acquisition of the PBMC sample; 5) age; 6) stage of acute phase/remission; 7) course of relapse; 8) the number of spinal cord segments involved by the lesion in magnetic resonance imaging (MRI) T2 weighted imaging (MRI‐T2); 9) count of brain lesions in MRI‐T2; 10) whether the lesion involves the optic nerve in MRI‐T2; 11) whether the lesion involves the spinal cord in MRI‐T2; 12) Expanded Disability Status Scale (EDSS), when sampling; 13) maintenance therapy; and 14) percentage of B cells in peripheral blood (Table S1, Supporting Information). Annualized relapse rate (ARR) was significantly correlated with EDSS and the number of spinal cord lesions in MRI‐T2 (**Figure** **1**A,B). We grouped all samples into high/low ARR groups according to median ARR (0.24), and the two groups were significantly different in terms of recurrence probability (Figure 1C). PBMC was isolated from each enrolled patient with NMOSD, followed by RNA extraction, mRNA sequencing, and mapping of reads to the human genome. Finally, the count matrix was standardized using the method of transcripts per kilobase of exon model per million mapped reads (TPM).

### 2.2 Developing Cuproptosis Signature (CS) and Validation

Cuproptosis genes were obtained based on a previous study^[^ ^7^, ^13^ ^]^ (see Supporting Information for details). Signal sample gene set enrichment analysis (ssGSEA) was performed on the Cuproptosis gene set, followed by the calculation of a CS for each sample that was related to the expression pattern of Cuproptosis genes. CS was significantly positively related to brain lesion counts in MRI‐T2 (Figure 1D) and positively related to the lesion counts of the spinal cord in MRI‐T2 (Figure 1E). This result indicates that CS is correlated with the severity of the disease. Additionally, CS was positively correlated with EDSS (Figure 1F), indicating that patients with higher CS had a worse prognosis. We also compared the MRI‐T2 lesion on the optic nerve with a CS, and the results showed that patients with optic nerve lesions obtained higher scores. Interestingly, CS also showed significant differences between HC and patients (Figure 1H). These results revealed the potential correlation that CS would be positively correlated with the processing and severity of the disease.

**Figure 2:**
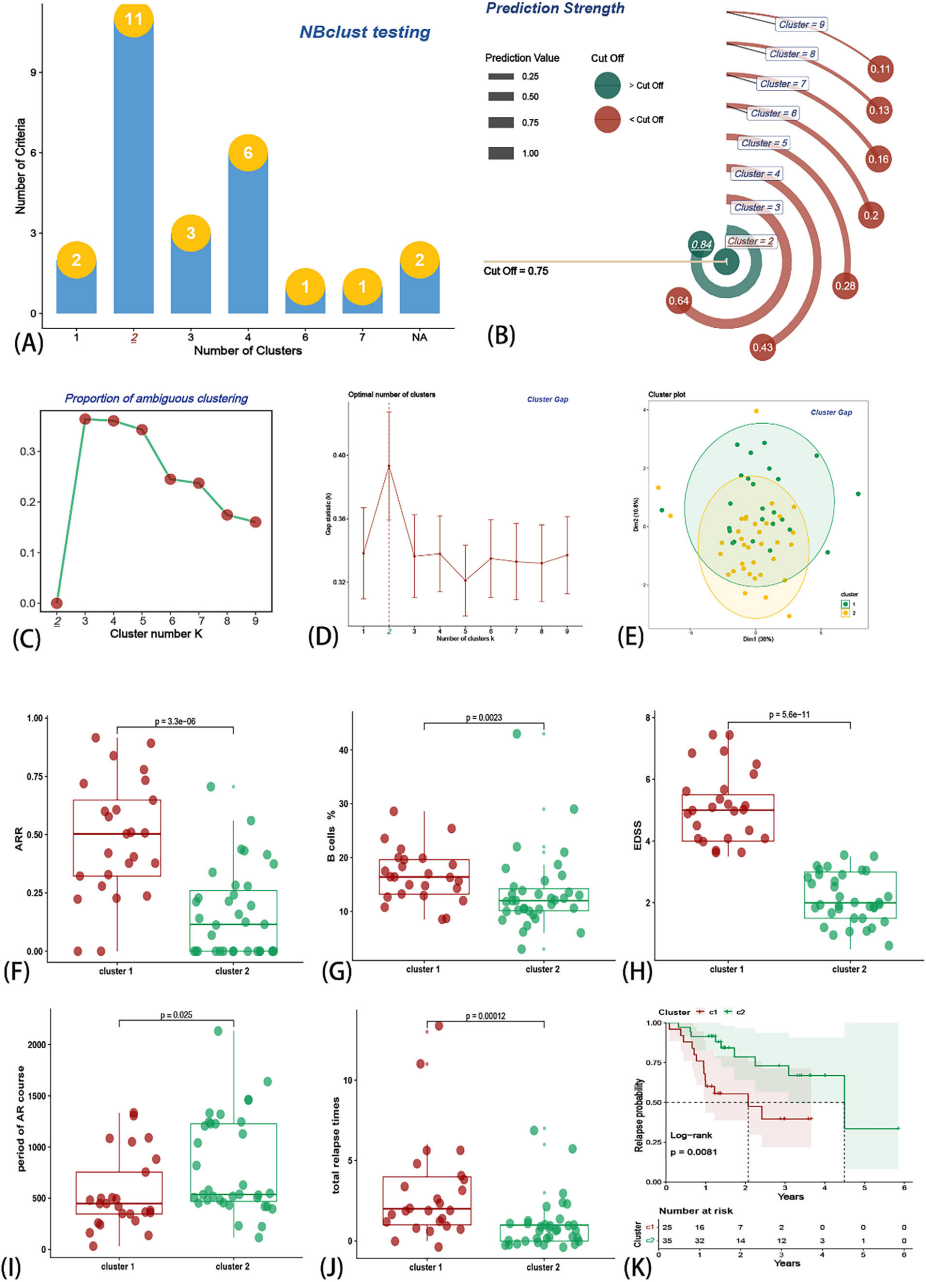
Evaluation of consensus clustering algorithms. Validation algorithms of consensus clustering algorithms showed optimal k = 2, NBclust testing algorithm (A), prediction strength algorithm (B), PAC algorithm (C), cluster gap algorithm (D,E). The following clinical traits showed significant differences between the two clusters: ARR (F), percentage of B cells (G), EDSS (H), the period of acute‐remitting (AR) course (I), total relapse times (G), and relapse probability (K).

Subsequently, we performed a consensus cluster analysis in which all NMOSD samples were initially divided into 2−9 clusters (k = 2, shown in Figure 1I). An optimal cluster was obtained when k = 2, according to consensus cumulative distribution function (CDF) (Figure 1J). In addition, we tested Nbclust (**Figure** **2**A), prediction strength algorithm (Figure 2B), proportion of(ambiguous clustering (PAC) statistics (Figure 2C), and cluster gap algorithm (Figure 2D,E). The same result (optimal cluster k = 2) was achieved using the 4‐individual clustering algorithm, which indicated the stability and robustness of the CS with consensus cluster algorithms. Cluster 1 was significantly higher than Cluster 2 in ARR (Figure 2F), B cell % (Figure 2G), EDSS (Figure 2H), and total recurrence times (Figure 2J), but lower in the total course (Figure 2I). These results indicate that the consensus cluster algorithm can divide patients with NMOSD into two clinically prognosis‐related groups. Patients in Cluster 1 had a worse prognosis and more serious symptoms. Meanwhile, the KM analysis also showed that patients in Cluster 1 were more likely to have a shorter recurrence period (Figure 2K).

Based on the ssGSEA and unsupervised clustering results, the CS demonstrated satisfactory reliability, which was closely related to important clinical signatures of prognosis and severity. Therefore, we believe that the physiological process of Cuproptosis is involved in the progression of NMOSD.

### 2.3 Identifying CS‐Related Modules Followed by Screening of Hub Genes

**Figure 3:**
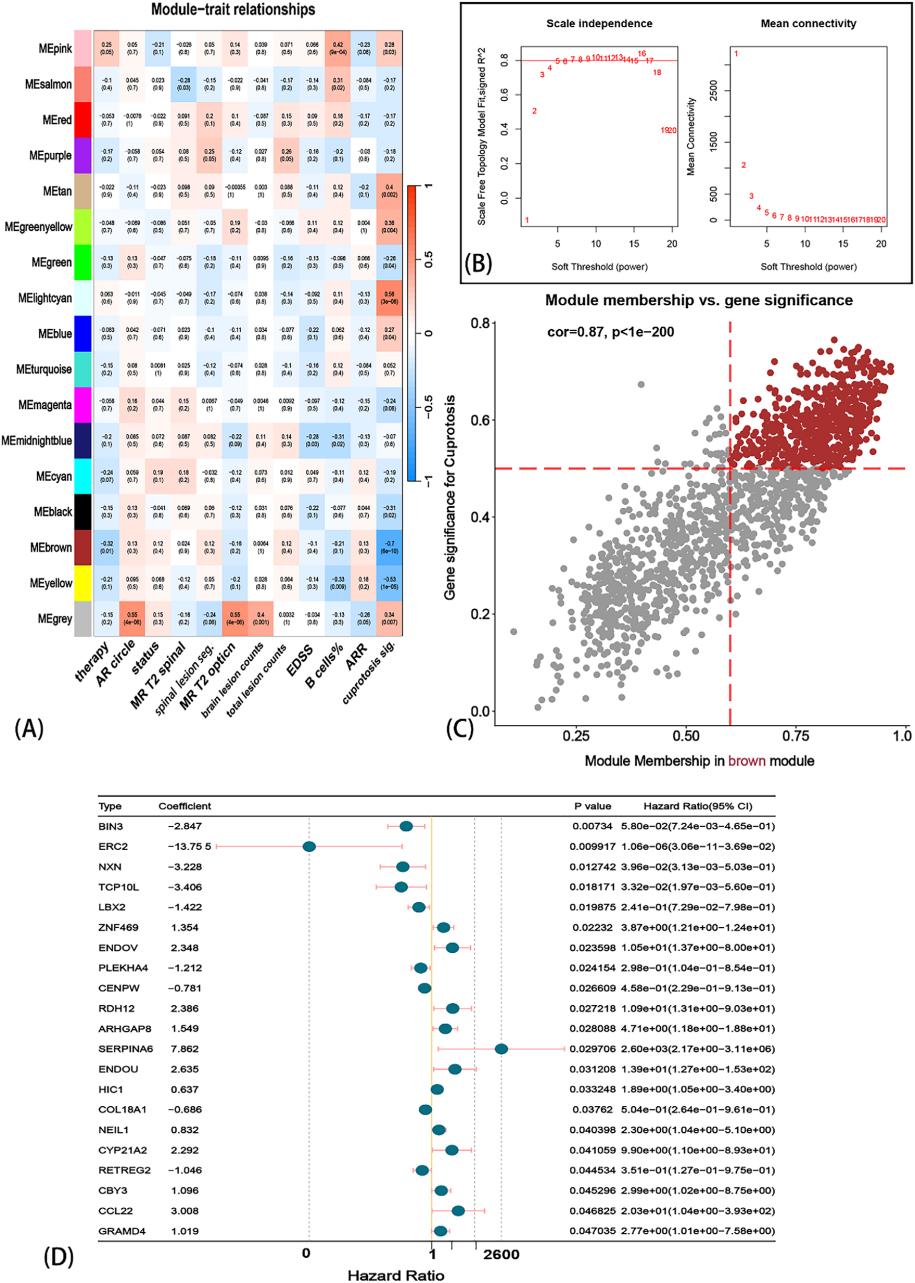
Screening Cuproptosis‐related‐prognosis (relapse) hub genes. Through WGCNA analysis, modules were calculated relationship with traits (A), when λ = 7 met the criterion of scale‐free network (B), the strongest correlation among module and traits was brown and Cuproptosis signature (CS), and the gene significance (GS) and module membership (MM) showed a satisfied correlation (cor = 0.87 and *p*‐value < 1e^−200^), the hub genes were screened by the criteria of GS > 0.5 and MM > 0.6 (C); univariate Cox regression was used to screen the relapse‐related genes based on *p* < 0.05 (D). Finally, 21 genes were obtained.

Weighted gene co‐expression network analysis (WGCNA) was performed using the mRNA‐seq matrix of patients with NMOSD. The soft threshold β was set at 7 when scale‐free R^2^ > 0.8, which could provide a suitable power value for the construction of the co‐expression network (**Figure** **3**B). Then, 17 modules were generated and are indicated in different colors (Figure 3A). Furthermore, the correlations between modules and key clinical traits, including maintenance therapy, AR‐course period, remission stage, MRT‐T2 spinal cord lesions, counts of MRT‐T2 spinal cord lesions, MRI‐T2 optic nerve lesions, counts of MRI‐T2 brain lesions, total counts of MRI‐T2 lesions, EDSS, B cell %, ARR, and CS, were calculated (Figure 3A). The strongest correlation was observed between the CS and the brown module. Furthermore, the dendrogram and heat map demonstrate the adjacency of the modules (Figure S1A, Supporting Information). In the brown module, the correlation coefficient between module membership (MM) and gene significance (GS) reached 0.87, with a *p*‐value less than 1×e^−200^ (Figure 3C). These results suggested that the quality of the Cuproptosis‐related module was superior. We identified the hub genes in the brown module, considering GS > 0.5 and MM > 0.6, and finally obtained 638 genes.

### 2.4 Integrative Machine Learning of the CRPR Model

**Figure 4:**
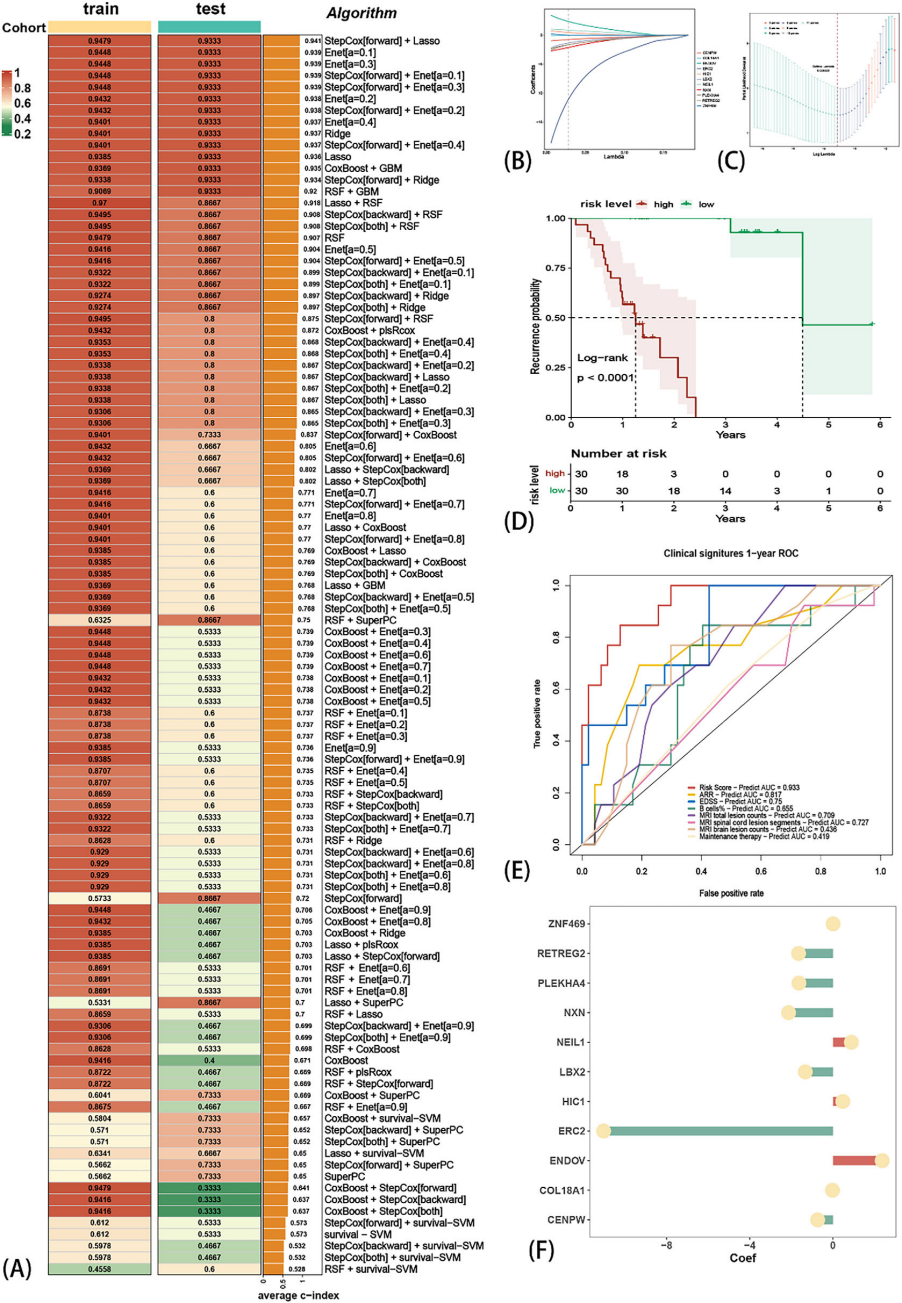
The consensus Cuproptosis‐related prognosis risk (CRPR) model was fitted through the machine learning algorithms integrative procedure and Leave‐One‐Out Cross‐Validation (LOOCV) framework. A total of 108 combinations of machine learning algorithms generated the C‐indices between the training cohort and testing cohort (A). The combination of StepCox (direction = forward) + LASSO showed the highest C‐index (CPRP model), which was followed by screening 11 genes by the minimum λ of this model (B,C). Based on this model, all patients were divided into two risk groups, and the relapse probability was significantly different between low‐ and high‐risk groups (D). The ROC showed the AUC of the CRPR model and other clinical traits (E). The coefficient of the CRPR model with 11 genes (F).

Based on the 638 Cuproptosis‐related hub genes we obtained from WGCNA, a univariate Cox regression algorithm was used to identify 21 genes with a *p*‐value < 0.05 (Figure 3D), followed by exclusion of the much lower expression value genes (*SERPINA* *6*) in all samples. Finally, 20 CS‐related genes were subjected to an integrative machine learning procedure to train the CRPR model. Stratified sampling was conducted to divide the 60 samples into training (48 samples) and testing cohorts (12 samples). In this part of the analysis, we selected the duration of the AR course and the status of recurrence as observation variables. We fit the models based on the Leave‐one‐out Cross‐Validation (LOOCV) framework. Then, 108 algorithm combinations were fitted in parallel to the training/test cohort. Therefore, our objective was to reveal the potential relationship between CS‐related genes and clinical prognosis. Furthermore, we calculated the C‐index of each combination in the training and test cohorts, followed by the generation of an average C‐index between the training and test cohorts (**Figure** **4**A). Interestingly, the highest average C‐index was obtained with the combination of StepCox (forward direction) and lasso (0.941). Using this combination, we first performed StepCox (direction = forward) and screened for significant genes (Figure S1B, Supporting Information), followed by LASSO regression. The optimal λ was observed when the partial likelihood of deviation reached the minimum value (Figure 4B,C). Finally, this combination generated a set of 11 genes and their coefficients (Figure 4F), followed by the calculation of a prognosis‐related model of the risk score for each sample, the CPRP model.

### 2.5 Evaluating the CRPR Model

**Figure 5:**
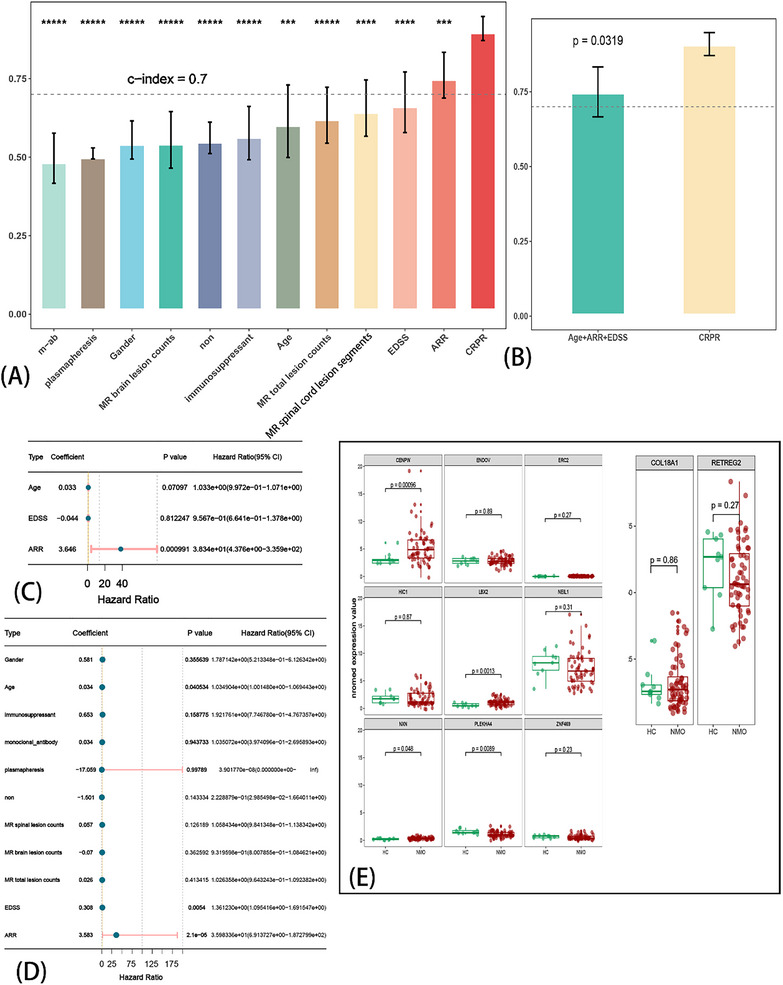
Validation of the Cuproptosis‐related prognosis risk (CRPR) model. The C‐index was compared between the CRPR model and other clinical traits via univariate Cox regression (A). The C‐index was compared between the CRPR model and prognosis (relapse) related traits (age, ARR, EDSS) through multivariant Cox regression (B). The forest plot showed multivariant Cox regression with age, ARR, and EDSS (C). The independent prognosis (relapse) risk factor screened by univariate Cox regression (D). The expression of 11 genes in the CRPR model between HC and NMOSD (E). ^***^ *p* < 0.001, ^****^ *p* < 0.0001, ^*****^ *p* < 0.00001, m‐ab = monoclonal antibody.

Based on the median risk score, we divided all samples into high‐ and low‐risk groups. The results of the KM analysis showed that patients in the high‐risk group had a significantly shorter recurrence period (Figure 4D). Furthermore, we performed the receiver operating characteristic (ROC) (Figure 4E) analysis to measure the discrimination of the CRPR model for the Area Under the Curve (AUC) of 1 year (0.954) and compared key clinical traits, including ARR (0.75), EDSS (0.817), the percentage of peripheral blood B cells (0.655), the counts of MRI‐T2 spinal cord lesions (0.547), the counts of MRI‐T2 brain lesions (0.547), the total counts of MRI‐T2 lesions (0.547), and the maintenance therapy (0.547). We also compared the C‐index of the CRPR with clinical traits (**Figure** **5**A). Only the C‐indices of ARR and CRPR were > 0.7, and CRPR was significantly higher than the other clinical traits. We selected clinical traits with a *p*‐value < 0.05 via univariate Cox regression (Figure 5D), followed by the construction of a multivariate Cox regression model of age, ARR, and EDSS (Figure 5C). The CPRP C‐index was also significantly higher than that of the multivariate Cox regression analysis for age, ARR, and EDSS (Figure 5B). The normalized expression of the 11 genes between healthy controls (HC) and NMOSD is also shown (Figure 5E). These results suggest that the CRPR model is robust, reliable, and precise in prognosis prediction. Therefore, CRPR can serve as an independent prognostic risk factor.

**Figure 6:**
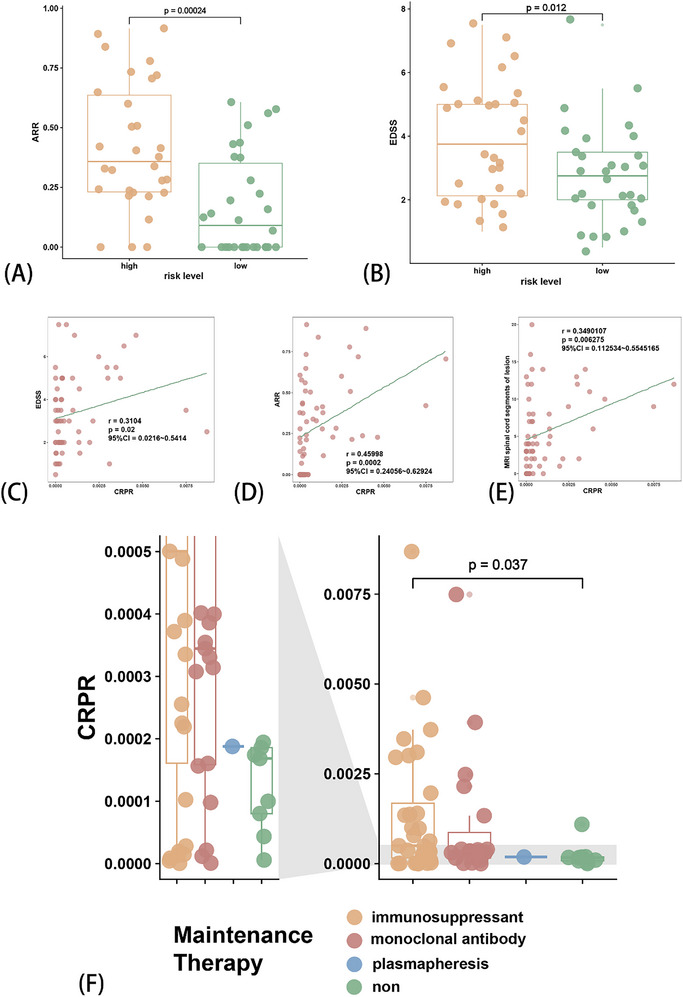
Accessing the Cuproptosis‐related prognosis risk (CRPR) model with clinical traits. Comparison of clinical traits between risk levels in the CRPR model: ARR (A) and EDSS (B); Comparison of clinical traits in the CRPR model: EDSS (C), ARR (D), counts of spinal cord lesions on MRI T2 weighted image (E), and the maintenance therapy (F).

Therefore, we compared other clinical traits (independent prognostic factors) using the CRPR model. Patients in the high‐risk group had higher ARR and EDSS scores (*p* < 0.05) (**Figure** **6**A,B). The risk score was also positively correlated with the EDSS (rho = 0.3104, p = 0.02, Figure 6C) and ARR (rho = 0.46, p = 0.0002, Figure 6D). Furthermore, the risk score showed a positive correlation with lesion counts in the spinal cord MRI T2 (Figure 6E). Moreover, the risk score was statistically significant when patients received immunosuppressants (Figure 6F). These results indicated that CRPR is clinically related and could potentially be a prognostic factor.

### 2.6 Developing of AQP4‐IgG^+^ NMOSD Recurrence Predicting Tools and Validation

**Figure 7:**
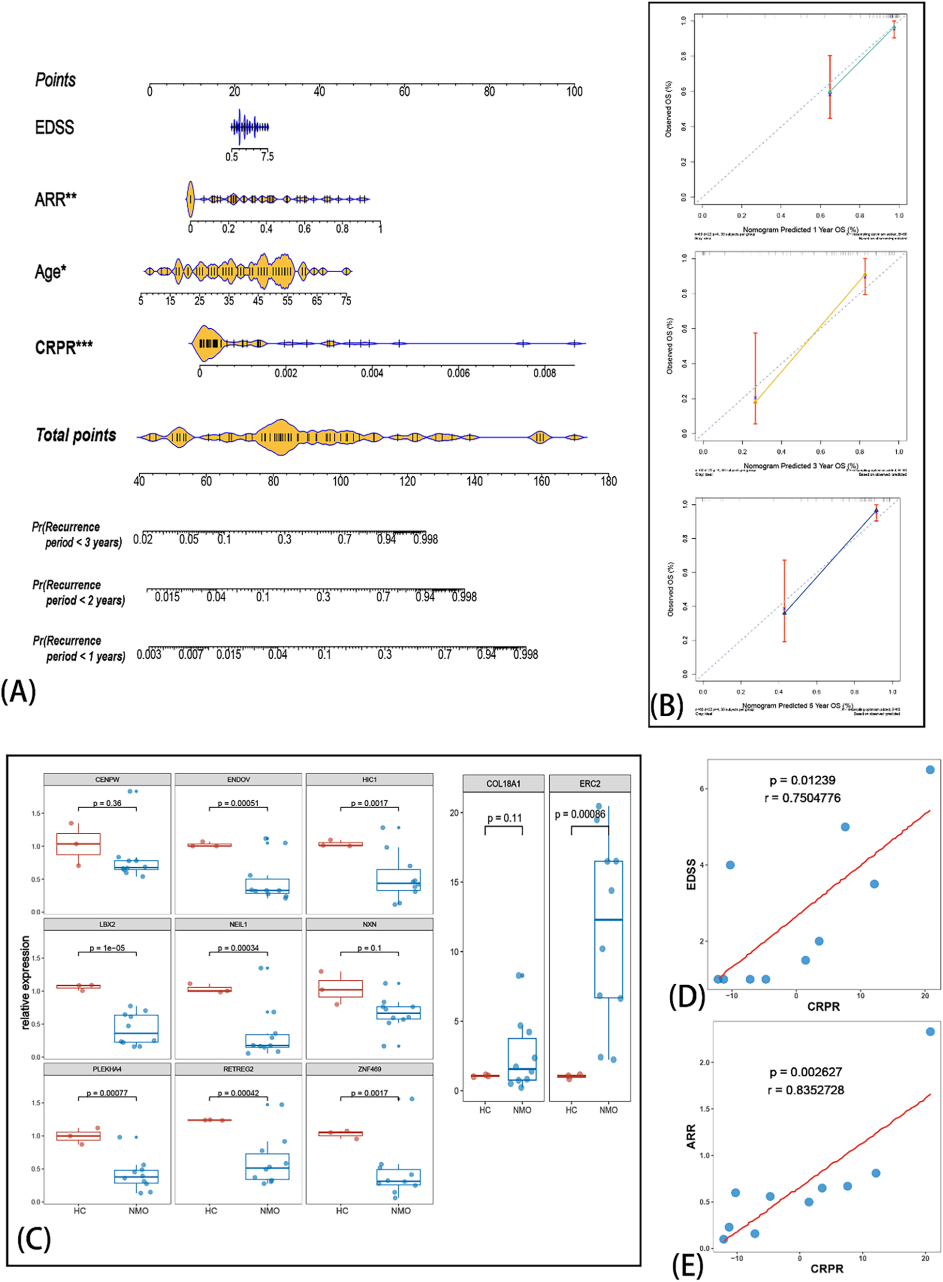
Development of a nomogram based on Cuproptosis‐related prognosis risk (CRPR) and independent prognosis (relapse)‐related risk factors, followed by external independent validation. The prognosis‐related risk nomogram was developed using CRPR and traits screened by univariate Cox regression (A). The calibration curve of a nomogram predicting 1, 2, and 3 years. RT‐PCR of PBMC in another 10 patients with NMOSD and three healthy controls (HC) revealing the related expression of 11 genes between NMOSD and HC (C). The calculated CRPR with validation cohort shows significant correlation with EDSS and ARR. ^*^ *p* < 0.05, ^**^ *p* < 0.01, ^***^ *p* < 0.001.

To resolve the issue of unpredictable recurrence mentioned in the introduction, we hoped to further exploit the implications of the CRPR model. Therefore, we constructed a nomogram with CRPR and statistically significant variations in the multivariate Cox regression analysis of clinical traits (Figure 5C). The nomogram could predict a 1/2/3‐years’ period of recurrence (**Figure** **7**A). The calibration curves for 1/2/3‐years showed good performance in terms of predicted reliability (Figure 7B).

Furthermore, we included another 10 patients with NMOSD (AQP4‐IgG^+^) into a validation cohort using the same criteria as those for the training cohort. The PBMC was obtained, followed by reverse transcription‐Polymerase Chain Reaction (RT‐PCR) with the 11 genes (see Table S2, Supporting Information for details of primer) of the CRPR model, and three HC PBMC samples were also included. The relative expression of the 11 genes was compared with that in the HC group (Figure 7C). The results indicated that a majority of the 11 genes showed statistically significant differences between the NMOSD and HC groups. The CRPR model was fitted to a validation group. Next, we calculated the relationship between EDSS (Figure 7D) and ARR (Figure 7E). The data indicated that CRPR had a higher correlation with ARR (cor = 0.7504, p = 0.0123) and EDSS (cor = 0.8353, p = 0.0026).

**Figure 8:**
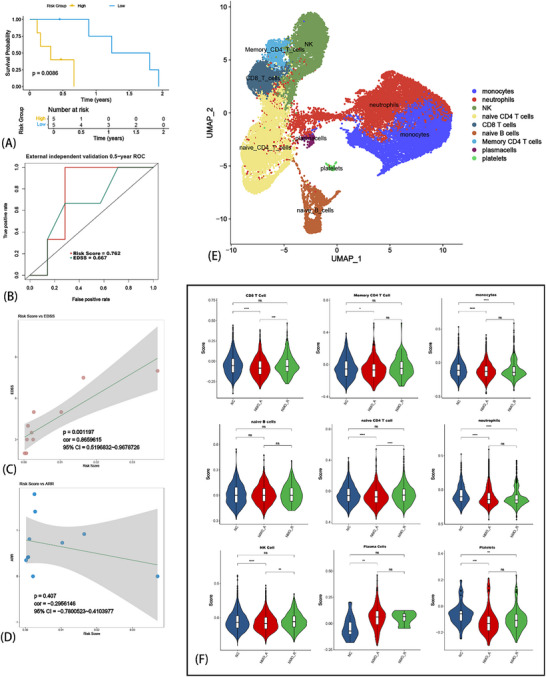
External validation of CRPR model and exploratory analysis of Cuproptosis in NMOSD relapse. Relapse‐free survival in low‐ versus high‐risk groups from multicenter validation cohort (log‐rank p = 0.0086) (A). Time‐dependent ROC curve for relapse prediction of risk score (AUC = 0.762 at 6 months) (B). Positive correlation between CRPR risk scores and EDSS (cor = 0.866, p = 0.0012) (C). Non‐significant trend between risk scores and ARR (cor = ‐0.296, p = 0.407) (D). UMAP projection of 9 peripheral blood immune subsets identified by scRNA‐seq (E). Gene set score of Cuproptosis across 9 cell subtypes via AddModuleScore algorithm (F). ^ns^ *p* > 0.05, ^*^ *p* < 0.05, ^**^ *p* < 0.01, ^***^ *p* < 0.001, ^****^ *p* < 0.0001.

We further validated the CRPR model in an independent multicenter cohort (n = 10, AQP4‐IgG^+^). Using identical preprocessing and scoring protocols, the model demonstrated satisfied predictive accuracy (AUC = 0.762, **Figure** **8**B). Patients stratified by CRPR risk scores showed significantly different relapse probabilities (log‐rank p = 0.0086; Figure 8A). Higher risk scores correlated with increased disability (EDSS: cor = 0.866, p = 0.0012; Figure 8C), while showing a non‐significant inverse trend with annualized relapse rate (ARR: cor = ‐0.296, p = 0.407; Figure 8D). This ARR finding may reflect the fact that some newly diagnosed cases (acute phase) in this cohort had a shorter clinical course with only one or two relapses, and others had a shorter follow‐up duration.

Although both internal and external validations support CRPR's predictive utility, further large‐scale prospective studies with extended follow‐up periods are warranted to establish clinical applicability.

### 2.7 Exploratory Single‐Cell Profiling Reveals Cuproptosis Pathway Enrichment in NMOSD T Cells

As a preliminary exploration of potential mechanisms underlying CRPR's predictive capacity, scRNA‐seq was conducted on whole peripheral blood from acute NMOSD (n = 2, AQP4‐IgG^+^), remission (n = 1, AQP4‐IgG^+^), and healthy control (n = 1) subjects matched to our mRNA‐seq sampling protocol. Based on the expression characteristics of cell markers, we performed dimensionality reduction using UMAP and identified 9 cell subsets (Figure 8E). Using the “AddModuleScore” algorithm in the *Seurat* package, we calculated the Cuproptosis gene set scores for each cell subset (Figure 8F). Notably, focusing on the process of NMOSD relapse (rather than the development of new‐onset NMOSD), we found that the Cuproptosis score in naïve CD4^+^ T cells was the most significantly reduced compared to other cell subsets (Figure 8E, naïve CD4^+^ T cell). This result suggests that Cuproptosis may be involved in the immune regulatory mechanisms of NMOSD relapse by affecting the differentiation of naïve CD4^+^ T cells. It should be emphasized that this study focuses on NMOSD relapse rather than new‐onset disease; thus, although some cells showed differences in Cuproptosis scores between the NMOSD group and healthy controls (HC), these were not the focus of our study. In addition, statistically significant reductions in Cuproptosis were observed in CD8^+^ T cells and NK cells between the acute and remission phases of NMOSD (Figure 8F, CD8^+^ T cell, NK cell). These findings indicated that: 1) the mechanism by which Cuproptosis affects NMOSD relapse may involve other alternative pathways; 2) due to the limitations of high‐throughput sequencing and statistical algorithms, differential results do not always reflect true molecular biological mechanisms and should be comprehensively interpreted in combination with physiological and pathological contexts.

In this study, we aimed to develop a Cuproptosis‐based CRPR model for predicting NMOSD relapse. The specific role of Cuproptosis in this process requires dedicated systematic laboratory research. Our future dedicated studies will elucidate Cuproptosis‐mediated mechanisms in NMOSD relapse using integrative molecular, immunological, and in vivo approaches. This work would provide a solid theoretical foundation for the CRPR model.

## 3 Discussion

Relapse plays an important role in NMOSD.^[^ ^1^, ^2^ ^]^ As a distinct characteristic, each relapse causes severe accumulated sequelae in patients with NMOSD,^[^ ^1^ ^]^ which negatively impacts the patients. However, little research has been conducted on this topic. Using a combination of machine learning algorithms, a CRPR model was developed and showed a high correlation with univariate/multivariate Cox regression to identify independent prognostic clinical risk signatures (age, ARR, and EDSS). Based on our systematic analysis, CRPR can be considered an independent clinical prognostic risk factor.

Cuproptosis, a copper‐induced cell death, has been observed in many diseases, such as Menkes disease,^[^ ^14^ ^]^ Wilson disease,^[^ ^15^ ^]^ Alzheimer's disease,^[^ ^16^ ^]^ rheumatoid arthritis,^[^ ^8^ ^]^ and some types of cancers.^[^ ^5^ ^]^ In our study, we did not perform a differential expression analysis of Cuproptosis‐related genes because Cuproptosis is not induced by a single gene but by an expression pattern of multiple genes.^[^ ^7^, ^17^ ^]^ Therefore, we hoped to develop a more suitable method to study how Cuproptosis affects the process of NMOSD. Consequently, the ssGSEA algorithm was used to generate the CS. A consensus clustering algorithm was used to verify the robustness of the CS patterns. With consensus clustering, two clinical prognosis‐related clusters were generated, and the results showed satisfactory consistency in correlation with clinical prognostic traits, which was compared to CS. We combined the above results with WGCNA to identify prognosis‐related hub genes, followed by an integrated machine‐learning combination algorithm. An integrated pipeline was developed to generate a consensus CRPR model. In total, 108 combinations of algorithms fit each model using the LOOCV framework. Due to the lack of available or suitable RNA‐seq datasets for patients with NMOSD in previous studies, we performed self‐validation by separating the test cohort from the 60 samples using stratified sampling. This integrative procedure benefited from fitting a model with consensus performance on the relapse‐related prognosis of NMOSD using a combination of various machine learning algorithms. Based on large‐scale combinations, the dimensionality of variables was reduced; the fitted model was more robust after comparing the C‐index; and more algorithmic combinations provided more possibilities for a result that was closer to the real situation. As NMOSD is a rare disease worldwide,^[^ ^1^ ^]^ limited samples of NMOSD can be acquired. Additionally, to obtain consensus and high‐quality data, patients with NMOSD who were not first diagnosed and did not receive treatment at the First Affiliated Hospital of Zhengzhou University throughout the process were also excluded from this study. Consequently, a larger number of samples were used to fit the model. However, our dataset was slightly smaller than the cancer dataset. To avoid instability and overfitting of the CRPR model, we perform a combination of 10 survival‐related machine learning algorithms. The strength of integrative algorithms is to fit the model with consensus performance on the prognosis of the CRPR model based on a variety of machine learning algorithms and their combinations, and algorithm combinations can further reduce the dimensionality of variables, making the model more simplified and translational. Our systematic evaluation of 108 combinations prioritized generalizability and selected Stepwise Cox (forward) followed by LASSO algorithm, for the highest validation C‐index, balanced feature genes selection, clinical interpretability of Stepwise Cox, and statistically significant correlation with clinical signature. The degradation between training and testing cohorts showed less than 0.1 (StepCox [forward] + LASSO combination, Δc‐index = 0.0146), which suggested that the performance drop satisfies the criterion for generalizability.^[^ ^18^ ^]^ This result represented robust generalization. Besides, we also collected another 10‐validation NMOSD samples (in‐house) and an independent validation cohort (10 samples) as a multi‐center cohort (from the First Affiliated Hospital of Xinxiang Medical University, Department of Neurology) that met the same criteria but were collected beyond the period of the previous 60 RNA‐seq samples. For validation, we first performed RT‐PCR on the PBMC of the in‐house validation cohort, followed by the calculation of the CRPR. Next, we collected an external independent validation cohort via identical pre‐treatment sampling followed by mRNA‐seq, harmonized clinical metadata, and uniform sequencing/normalizing protocols. The CRPR model also showed good performance via an external independent validation cohort. The CRPR model maintained high accuracy, consensus, and stable performance with an in‐house and external independent validation cohorts, displaying satisfactory potential for clinical application. Given NMOSD's rarity (incidence ≤0.73/100000 person‐years), our 83‐patient multicenter cohort provides meaningful evidence for CRPR's predictive potential. While this sample size exceeds most NMOSD‐related studies, we emphasize that clinical deployment requires validation in larger, prospective cohorts with diverse ancestries. Planned integration with the multicenter collaboration's registry (target n = 500) will further assess real‐world utility.

Our model's applicability is confined to AQP4‐IgG antibody positive NMOSD, reflecting the cohort's intentional focus on this pathophysiologically defined subgroup. Generalization to AQP4‐IgG antibody negative disease is not supported at this stage, as emerging evidence suggests these may represent separate disorders^[^ ^19^ ^]^ (e.g., MOGAD). Whether the CRPR model applies to AQP4‐IgG antibody negative NMOSD still requires further dedicated investigation.

Conventional clinical traits, such as ARR, EDSS, and counts of lesions in the spinal cord and brain, are recorded to evaluate disease progression.^[^ ^20^ ^]^ Although AQP4‐IgG is considered a biomarker of NMOSD, there are still some cases of AQP4‐IgG antibody negative but NMOSD‐like syndromes.^[^ ^20^, ^21^ ^]^ The rate of seropositivity of AQP4‐IgG could be affected by an unsuitable period for collecting peripheral blood, the limitations of medical laboratory examination technology and methods, or other unclear disease statuses. Therefore, some NMOSD cases cannot be definitively diagnosed until secondary release. In addition, biomarkers such as AQP4‐IgG or other clinical traits alone cannot diagnose NMOSD. In our study, we found that some clinical traits, including age, ARR, and EDSS score, were correlated with prognosis and independent risk factors for relapse. However, interestingly, the C‐index of univariate Cox regression in MR spinal cord lesion counts appeared higher than that in age, but the *p*‐value was higher. Usually, the C‐index is the AUC of an ROC that plots the sensitivity against 1 minus specificity and stability of the model,^[^ ^22^ ^]^ but the *p*‐value often regards the contribution of the variate. In this case, MRI spinal cord lesion counts were recorded at the time point of examination; however, age can reflect the current status of the patient. Therefore, the model of MR spinal cord lesion counts appeared more stable than that with age; however, age showed a greater contribution to relapse. This contradiction is easy to understand. Interestingly, we found that the notable signature of CRPR worked independently of those factors but had significantly better performance in predicting relapse probability. Even in the multivariable Cox regression analysis of age, ARR, and EDSS, CRPR showed superior performance. As we fitted the CRPR model by sequentially reducing the dimensionality with two different machine learning algorithms, this model offers desirable extrapolation possibilities.

As previously mentioned, NMOSD is a rare disease. We also collected 10 samples to assess and verify our previous findings. The results preliminarily demonstrate the reliability and availability of the CRPR. Although more than 71.6% of the participants had a follow‐up period of more than 3.1 years, the sample size and follow‐up period should be expanded to make this model more precise.

The immunomodulatory effect of T cells may further explain the potential mechanism by which apoptosis participates in the relapse of NMOSD. Several studies have demonstrated the contribution of T cells to the pathophysiology of NMOSD. T follicular helper cells also contribute to the progression of NMOSD through the activation and maturation of naïve B cells.^[^ ^23^ ^]^ Activation of aquaporin 4‐specific Th17 cells can generate a robust autoimmune inflammatory response, and the experimental disease is exacerbated.^[^ ^11^ ^]^ A recent study found that an imbalance in Th1/Th17 cells was associated with NMOSD activity and severity.^[^ ^24^ ^]^ Furthermore, altered naïve CD4^+^ T cell homeostasis can cause severe disease in NMOSD.^[^ ^25^ ^]^ The differentiation of T cells depends on^[^ ^26^ ^]^ naïve CD4 T, Th1,^[^ ^10^ ^]^ Th17,^[^ ^5^, ^27^ ^]^ and Treg^[^ ^28^ ^]^ cells, and the TCA cycle also targets the balance of Th1/Th17.^[^ ^28^, ^29^ ^]^ All these cells play an important role in the progression of NMOSD. Interestingly, as mentioned previously, Cuproptosis can target the TCA cycle.^[^ ^5^, ^7^ ^]^ Therefore, based on the evidence of Cuproptosis, this process may target the TCA cycle in T cells and skew their polarization. Finally, disrupting T cell homeostasis, such as the balance of Th1/Th17 cells, can also lead to relapse of NMOSD. However, the precise mechanisms linking Cuproptosis to NMOSD immunopathology remain incompletely defined. Our future work will systematically study this through a well‐constructed experimental design and comprehensive mechanistic investigations.

There are still limitations of this research: First, despite adding an external validation, the cohort size remains constrained by NMOSD rarity. Second, while the CRPR model predicts relapse, its biological basis in NMOSD pathogenesis requires experimental validation. Third, the validation cohort had shorter follow‐up; longer observation and more centers’ cohorts may refine risk thresholds. The CRPR model's strong performance opens key biological questions: Does elevated Cuproptosis directly drive NMOSD relapse, or is it a bystander effect? Resolving this requires several laboratory research, such as the correlation of CRPR scores with the cerebrospinal fluid (CSF) markers of Cuproptosis (e.g., ferredoxin‐1, lipoylated proteins); functional validation in AQP4‐IgG antibody positive patients of NMOSD immune cells; therapeutic trials of copper modulators in high‐risk subgroups. These mechanistic studies are beyond our predictive modeling scope but are prioritized in our subsequent research.

In conclusion, based on systematic bioinformatics analysis and multidimensional validation, we developed a robust and stable CS to assess the prognosis and generated a nomogram to predict the relapse of AQP4‐IgG antibody positive NMOSD. This CRPR model provides a promising direction to optimize the management of patients with AQP4‐IgG antibody positive NMOSD and develop advanced interventions to avoid NMOSD relapse.

## 4 Experimental Section

### Clinical Data Collection from Patients with NMOSD

All enrolled patients of training/testing and RT‐PCR validation cohorts were screened according to the 2015 international consensus‐based diagnostic criteria for NMOSD at the First Affiliated Hospital of Zhengzhou University Department of Neurology and received available standard systemic therapies according to the guidelines. The clinical signatures of age, sex, disease status (acute/remission phase), maintenance therapy (long‐term management) of the remission phase, disease course, overall relapse rate, last period of AR course, annualized relapse rate (ARR), latest magnetic resonance imaging (MRI) T2 results prior to the samples collection (the number of spinal cord segments involved by the lesion, brain lesion counts), Expanded Disability Status Scale (EDSS), and percentage of peripheral blood B cells were recorded. The external validation cohort was provided by the Department of Neurology, First Affiliated Hospital of Xinxiang Medical University (multi‐center), and followed the same procedures for sample collection, mRNA‐seq, and data analysis as the training/test cohort. Serum AQP4/MOG/GFAP‐IgG antibodies were quantified using a dual‐fluorescence cell‐based assay (CBA) with live HEK293 cells co‐transfected with human AQP4, MOG, and GFAP. This study was approved by the Ethics Committee of the First Affiliated Hospital of Zhengzhou University (No. 2024‐KY‐1904), and all enrolled patients have signed the informed consent forms.

### Screening of PBMC from Patients with NMOSD and Healthy Controls (HC)

PBMC (training cohort: 60 NMOSD vs 9 HC, RT‐PCR validation cohort: 10 NMOSD vs 3 HC, and external independent cohort: 10 NMOSD) was isolated using the standard procedure of “Ficoll” density gradient centrifugation with “Ficoll” reagent (abs930, Absin, Shanghai, China) and thermostatic centrifuge (8510R, Eppendorf, Germany). PBMC was collected and frozen in cryoprotectant (A2644601, Gibco, the United States) at −80 °C. Written informed consent was obtained from all enrolled patients, and the study was approved by the Ethics Committee of the First Affiliated Hospital of Zhengzhou University.

### PBMC RNA Isolation and Library Preparation

Total RNA was extracted using the TRIzol reagent (T9424, Sigma–Aldrich, Germany) according to the manufacturer's protocol. RNA purity and quantification were evaluated using the NanoDrop 2000 spectrophotometer (Thermo Fisher Scientific, MA, United States). RNA integrity was assessed using the Agilent 2100 bioanalyzer (Agilent Technologies, Santa Clara, CA, United States). Libraries were constructed using the VAHTS Universal V6 RNA‐seq Library Prep Kit, according to the manufacturer's instructions.

### mRNA Sequencing Process

Libraries were sequenced on an Illumina NovaSeq 6000 platform, and 150 bp paired‐end reads were generated. Raw reads in fastq format files were first processed using fastp1, and low‐quality reads were removed to obtain clean reads. Clean reads were mapped to the reference genome using HISAT22. The Transcripts Per Kilobase Million (TPM) of each gene was calculated, and the read counts of each gene were obtained using HTSeq‐count. Transcriptome sequencing and basic analyses were performed by OE Biotech Co., Ltd. (Shanghai, China).

### Cuproptosis‐Related Gene Signature Quantification Followed by Unsupervised Clustering and Validation

Single‐sample Gene Set Enrichment Analysis (ssGSEA) was performed to calculate the score of each NMOSD sample based on the expression value of the Cuproptosis gene set using the R package *GSVA*. Our objective was to verify the robustness of the ssGSEA results in the context of other clinical signatures. These ssGSEA results were significantly related to the prognosis. A consensus clustering algorithm from the *ConsensusClusterPlus* package was used to classify all NMOSD samples into several clusters based on Cuproptosis‐related genes, followed by a comparison of the differentiation of clinical signatures. The optimal number of clusters was verified by consensus matrix, Cumulative Distribution Function (CDF) curve, Proportion of Ambiguous Clustering (PAC) score, NBclust testing (by package *NBclust*), prediction strength (via package *fpc*), and gap statistics (by package *cluster*).

### Weighted Correlation Network Analysis (WGCNA)

The co‐expression network of mRNA in NMOSD‐PBMC was produced by the *WGCNA* package with an optimal soft threshold β that met the criteria for the scale‐free network. Additionally, a Topological Overlap Matrix (TOM) was generated using a weighted adjacency matrix, followed by the generation of the corresponding dissimilarity (1‐TOM). The Cuproptosis‐related module was used, and the hub genes inside this module were selected depending on Gene Significance (GS) and Module Membership (MM), both greater than 0.5.

### NMOSD Relapsing Course‐Based Machine Learning Integrative Procedure

To develop a Cuproptosis signature (CS) with high accuracy and stability, 10 machine‐learning algorithms were integrated, and obtained 108 algorithm combinations. These 108 algorithm combinations were classified according to the average concordance index (C‐index) between the training and testing cohorts. The 10 types of machine learning algorithms included CoxBoost (package *CoxBoost*), elastic network (Enet, package *glmnet*), generalized boosted regression modeling (GBM, package *gbm*), LASSO (package *glmnet*), partial least squares regression for Cox (package *plsRcox*), random survival forest (RSF, package *randomForestSRC*), supervised principal components (SuperPC, package *superpc*), stepwise Cox (package *stats* and *survival*), survival support vector machine (survival‐SVM, package *survivalsvm*), and Ridge (package *glmnet*). CS was generated by the following workflow: 1) screening prognosis‐related genes from WGCNA hub genes in the Cuproptosis‐related module by univariate Cox regression; 2) generating the training/validation (80%/20%) cohort by stratified sampling; 3) applying 108 algorithm combinations to the prognostic genes to fit prediction models based on the Leave‐One‐Out Cross‐Validation (LOOCV) framework^[^ ^30^ ^]^ in the training cohort; 4) reapplying all algorithm combinations in the validation cohort; 5) calculating the C‐index for each combination between the training and validation cohort, followed by generating the average C‐index for each combination; and 6) finally generating the CS using the optimal combination (combination with the highest average C‐index).

### Cuproptosis‐Based Clinical Signature Prediction Nomogram

Clinical signatures significantly related to prognosis were screened through univariate and multivariate Cox regression, followed by the combination of CS to develop a nomogram (package *regplot*). Calibration curves were constructed to validate the reliability of the nomogram (package *rms*).

### Reverse Transcription Quantitative Polymerase Chain Reaction (RT‐qPCR)

Total RNA was extracted with TRIzol (T9424, Sigma–Aldrich, Germany). Reverse transcription was performed using HiScript II Q RT SuperMix for qPCR (Vazyme, China). qPCR was performed using Taq Pro Universal SYBR qPCR Master Mix (Vazyme, China). The levels of HIC1, RETREG2, LBX2, ERC2, ZNF469, PLEKHA4, CENPW, NEIL1, ENDOV, COL18A1, NXN were normalized to GAPDH based on the 2^−ΔΔCt^ method. The primer sequences are shown in Table S2 (Supporting Information).

### Single Cell mRNA Sequencing (scRNA‐seq) and Data Analysis

Peripheral blood was prepared into a single‐cell suspension according to standard procedures. Then cells were loaded into a microfluidic chip of Chip A Single Cell Kit v2.1 (MobiDrop (Zhejiang) Co., Ltd.) to generate droplets with MobiNova‐100 (MobiDrop (Zhejiang) Co., Ltd). Each cell was involved in a droplet which contained a gel bead linked with up to millions oligos (cell unique barcode). After encapsulation, droplets suffer light cut by MobiNovaSP‐100 (MobiDrop (Zhejiang) Co., Ltd.) while oligos diffuse into the reaction mix. The mRNAs were captured by cell barcodes with cDNA amplification in droplets. Following reverse transcription, cDNAs with barcodes were amplified, and a library was constructed using the High Throughput Single‐Cell 3′ Transcriptome Kit v2.1 (MobiDrop (Zhejiang) Co., Ltd.) and the 3′ Dual Index Kit (MobiDrop (Zhejiang) Co., Ltd.). The resulting libraries were sequenced on an Illumina System according to the manufacturer's instructions(Illumina) at CHI BIOTECH CO., LTD. Fastp was used to perform basic statistics on the quality of the raw reads. Raw data (fastq format) of single‐cell 3′transcriptome was pre‐analyzed by MobiVision (version 3.2, MobiDrop), and reads were aligned to the reference GRCh38. Filtered cell‐gene matrix was obtained with MobiVision. Single‐cell sequencing bio‐information analysis was performed in R program with R package of *Seurat*, *edgeR*, and *clusterProfiler*, et al.

### Statistical Analysis and Plotting

All data processing, statistical analyses, and result plotting were performed using *R* (v. 4.4.1). The correlation between two observations was assessed using Pearson's correlation when the data were normally distributed; otherwise, Spearman's rank correlation was used. The t‐test and Wilcoxon rank‐sum test were used for continuous variables, and the chi‐square test was used for categorical variables. Cox regression analysis was performed using *survival* or *rms* packages. Kaplan–Meier (KM) analysis was performed using the *survival* package. The Cox regression result was displayed using the *forestplot* package. The receiver operating characteristic (ROC) curve was used to assess the relationship between the analytical methods specificity and sensitivity using the *survivalROC* package. The difference between the C‐indices of the signatures was assessed using the *CompareC* and *rms* packages. Statistical significance was set at *p*‐value < 0.05.

## Conflict of Interest

The authors declare no conflict of interest.

## Author Contributions

P.L., R.L., X.W., and H.L. contributed equally to this work and share first authorship. H.L., X.L., and B.Y. conceived this research. P.L., R.L., X.W., and H.L. performed the bioinformatics analysis and generated the figures and tables. P.L., R.L., H.L., H.H., A.S., W.Z., J.L., J.P., W.X., L.L., and H.J. collected PBMC and clinical data of the NMOSD samples. P.L., R.L., H.L., J.L., H.H., J.P., A.S., H.J., L.W., and D.C. performed the laboratory research. P.L., X.W., and R.L. wrote the manuscript. H.L., D.Y., X.L., and B.Y. revised the manuscript. All authors contributed to this work and approved the submitted manuscript.

## Supporting information



Supporting Information

## Data Availability

All clinical and sequencing data used in this study were based on an in‐house cohort and are available on request by emailing the corresponding author, H Liu (including R code).
