# Supplementary material for: Cuproptosis Signature Would Reveal the Acute‐Remitting Pattern in Patients with Neuromyelitis Optica Spectrum Disorder
Source: Adv Sci (Weinh). 2025 Aug 14;12(41):e17124. doi: 10.1002/advs.202417124 (PMC12591142; doi:10.1002/advs.202417124)
Supplement: Supplementary file 1 — Supporting Information [file ADVS-12-e17124-s001.docx]

S Figure 1


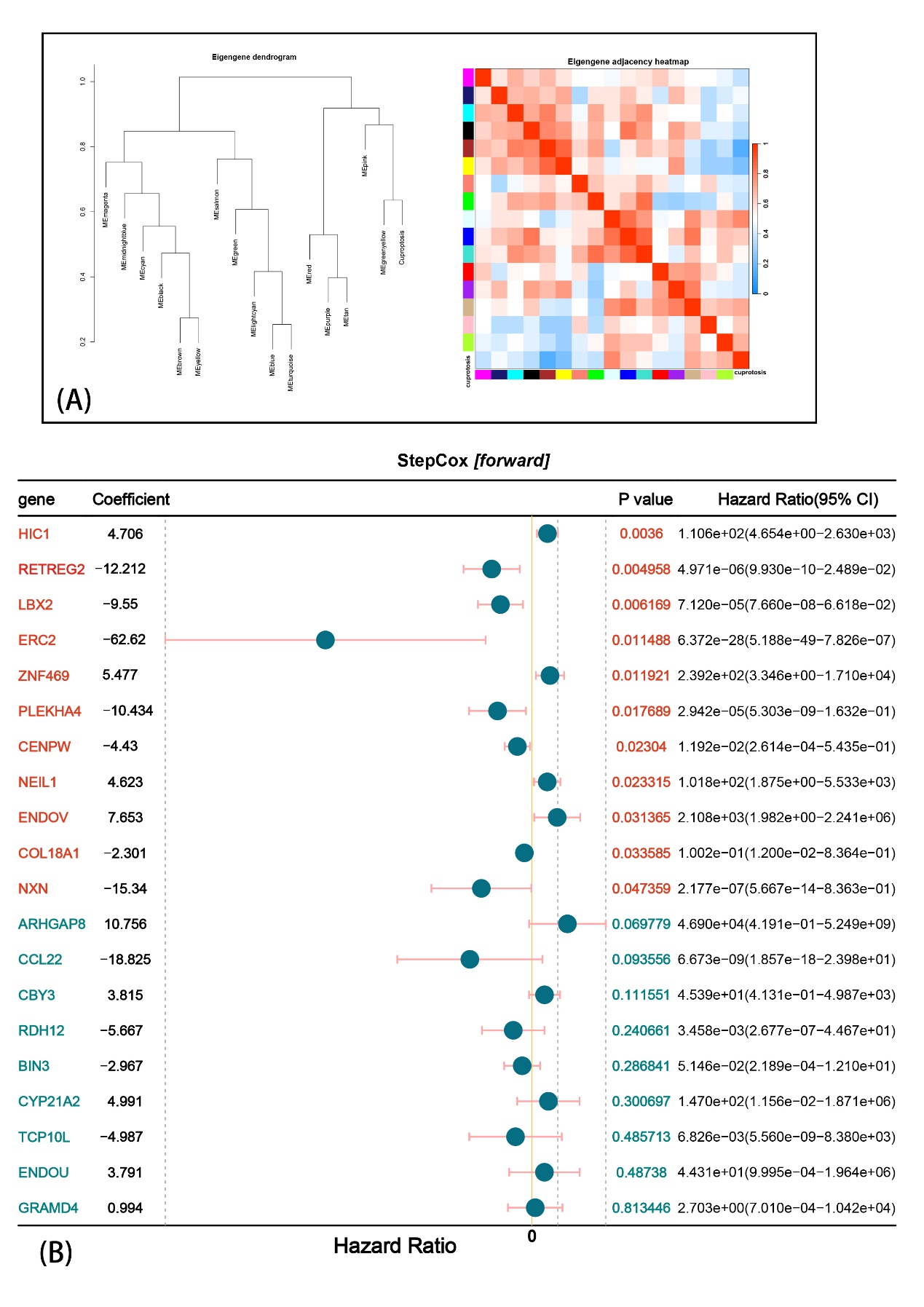


| Signature | ARR level | |
| --- | --- | --- |
|  | high | low |
| Age | 41.8 ± 14.1 | 40.1 ± 16.4 |
| Male | 4 | 7 |
| Female | 27 | 22 |
| Therapy-IS | 15 | 16 |
| Therapy-AB | 14 | 5 |
| Therapy-PM | 0 | 1 |
| Therapy-non | 2 | 7 |
| total Course (days) | 2405.2 ± 2469.1 | 1912.1 ± 1729.7 |
| Acute-remission course (days) | 151.3 ± 307.4 | 887.6 ± 503.1 |
| MRI spinal cord segments of lesion | 6.2 ± 4.5 | 4.7 ± 5.4 |
| MRI brain lesion count | 1 ± 2.1 | 2.8 ± 3.6 |
| MRI optic nerver + | 9 | 13 |
| MRI optic nerver - | 22 | 16 |
| MRI spinal + | 28 | 23 |
| MRI spinal - | 3 | 6 |
| EDSS | 4.3 ± 1.6 | 2.4 ± 1.4 |
| B cells % | 16.8 ± 7.3 | 12.7 ± 4.5 |

S Table 1.

Supplement table 1: Summary of key clinical signatures

IS = immunosuppressant; AB = monoclonal antibody; PM = plasmapheresis;

S table 2.

| Gene name | Forward Primer | Reverse Primer |
| --- | --- | --- |
| HIC1 | ACAAGAGCAGCAGCGAGGAG | CAGGTGCGGGCATGGGTAG |
| RETREG2 | TTGTCTTGTTGAGTATCCTGCTGTG | GCTTCTGCCTTCATGCTGTAGTC |
| LBX2 | AGCTTAGCACTGCCCGAAGG | GTCCACCTGTATCTCCTCGTCTG |
| ERC2 | GGAGGTGGAGAATGAGAAGAATGAC | ACTGTTGATTGTGCTTGAGGTTGG |
| ZNF469 | TTCTTCCACCCACCCACTCAC | GGCTGGCTCTGGCTGGTAG |
| PLEKHA4 | ATACGCTGCTGACCAAGTTGTG | GCTGCTTCTAGTTGCTCCTTCTC |
| CENPW | TGGTGACTTATTGGTCCATCTGAAC | TGACTCTACATTTACTCGCACAAGC |
| NEIL1 | GGCGGCTGCGTGGAGAAG | TGAAGCTGAGATGCGGTAGGC |
| ENDOV | AGCCACGACCGCAGCAC | GCAGCAGCAGCAAGTCAGG |
| COL18A1 | GAGTTCCAGAGAATGCCGCTTG | CCGCCTGAGCCACGAAGAG |
| NXN | GCGGGTGGAGGTGCTGAAC | CGGCGTTGGAGTCGGAGAG |
| GAPDH | TGACATCAAGAAGGTGGTGAAGCAG | GTGTCGCTGTTGAAGTCAGAGGAG |

Supplement table 2: The rt-PCR primer’s information of enrolled 11 genes
